# Altered interpersonal distance regulation in autism spectrum disorder

**DOI:** 10.1371/journal.pone.0283761

**Published:** 2023-03-31

**Authors:** Kinga Farkas, Orsolya Pesthy, Anna Guttengéber, Anna Szonja Weigl, András Veres, Anna Szekely, Eszter Komoróczy, Bálint Szuromi, Karolina Janacsek, János M. Réthelyi, Dezső Németh

**Affiliations:** 1 Department of Psychiatry and Psychotherapy, Semmelweis University, Budapest, Hungary; 2 Brain, Memory and Language Research Group, Institute of Cognitive Neuroscience and Psychology, Research Centre for Natural Sciences, Budapest, Hungary; 3 Doctoral School of Psychology, ELTE Eötvös Loránd University, Budapest, Hungary; 4 Institute of Psychology, ELTE Eötvös Loránd University, Budapest, Hungary; 5 Université Claude Bernard Lyon 1, CNRS, INSERM, Centre de Recherche en Neurosciences de Lyon CRNL U1028 UMR5292, Bron, France; 6 Obimon Systems Ltd., Budapest, Hungary; 7 MTA-ELTE Lendület Adaptation Research Group, Institute of Psychology, ELTE Eötvös Loránd University, Budapest, Hungary; 8 Centre for Thinking and Learning, Institute for Lifecourse Development, School of Human Sciences, Faculty of Education, Health and Human Sciences, University of Greenwich, London, United Kingdom; Universitat de les Illes Balears, SPAIN

## Abstract

Interpersonal distance regulation is an essential element of social communication. Its impairment in autism spectrum disorder (ASD) is widely acknowledged among practitioners, but only a handful of studies reported empirical research in real-life settings, focusing mainly on children. Interpersonal distance in adults with ASD and related autonomic functions received less attention. Here, we measured interpersonal distance along with heart rate variability (HRV) in adults with ASD, and tested the modulatory effects of eye-contact and attribution. Twenty-two adults diagnosed with ASD and 21 matched neurotypical controls participated in our study from October 2019 to February 2020. Our experimental design combined the modified version of the stop distance paradigm with HRV measurement controlling for eye contact between the experimenter and the participant to measure interpersonal distance. Still, we did not detect significant modulatory effect of eye contact and attribution. Our results showed a greater preferred distance in ASD. Moreover, we found lower baseline HRV and reduced HRV reactivity in ASD; however, these autonomic measurements could not predict preferred interpersonal distance. Our study highlights the importance of interpersonal space regulation in ASD: it might be considered that people with ASD need individually variable, presumably greater interpersonal distance. In addition, regardless of the distance they may have reduced autonomic regulatory capacity in social situations. Our results could help shape future experiments with sophisticated designs to grasp the complexity and underlying factors of distance regulation in typical and atypical populations.

## Introduction

Autism spectrum disorder (ASD) is a neurodevelopmental condition characterised by persistent difficulties in social communication and social interaction across multiple contexts, such as abnormal social approach or failure to initiate or respond to social interactions; and restricted, repetitive patterns of behaviour, interests, or activities [1]. At the neural level, cortical [2, 3], subcortical [4, 5], and autonomic [6, 7] neural alterations can be observed, including developmental, structural and functional differences [8–10] in parallel to the pervasive cognitive [11, 12], behavioural and physiological disturbances in ASD. However, one of the key components of social behaviour, namely interpersonal distance regulation, has received relatively less attention in ASD research (see exceptions: [13–16]) even though its impairment in ASD is widely acknowledged among practitioners. Our study aims to measure the interpersonal distance regulation and a related physiological parameter (heart rate variability, HRV) during this task and test the modulatory effect of two relevant factors in social communication: eye contact and attribution of self or the other in autism spectrum disorder.

Finding the appropriate social distance can be seen as the first step of physical social interactions. It is widely believed among practitioners that people with ASD keep a greater or abnormal distance [17] and violations of personal space also occur more often in ASD in childhood [18]. However, it is challenging to measure this phenomenon experimentally with high ecological validity, and the results are inconsistent. In autistic participants, preference for both closer [15, 19–21] or farther distance [16, 22, 23] can be found in the literature. Among these studies, only one measured interpersonal distance in adult ASD in real-life; they found no difference between study groups [18], an fMRI study found stronger feelings of discomfort in ASD when observing someone approaching them [14]. Studies applying electrophysiological or imaging methods usually present video recordings of an approaching individual [14] or use virtual reality displays [24–26] to measure interpersonal distance regulation in ASD. We argue that virtual displays might be useful in training or therapeutic settings, but they cannot take into account all the sensory modalities (e.g. external: olfactory information, nonverbal acoustic cues such as shuffling, sighing, croaking, coughing; internal: proprioception, and kinaesthesis), and the awareness of the presence of another person in the room. Furthermore, VR settings do not require mutual and real social interaction: participants do not need to consider the effect of their own presence on the other person while measuring the behavioural and physiological reactions in interpersonal interactions. In the present study, we measured the interpersonal distance among adult participants with ASD in an experimental setting with personal presence as close as possible to real-life situations.

Despite its relevance, empirical studies on interpersonal distance regulation of participants with ASD were conducted only in the past few years. The nomenclature of the concept is still not unified. *Personal space* [16, 19, 22], *social* or *physical distance* [18], and *interpersonal space* and *-distance* [13, 14, 16] are all commonly used. When measuring the physical distance between two people in one dimension, we use the term *interpersonal distance*. The stop distance paradigm [27] is the most commonly used method for measuring interpersonal distance regulation. This method is considered an ecological measure of permeability and flexibility of interpersonal space regulation [13]. The only study using the Stop Distance Paradigm among adults found no difference between groups in terms of interpersonal distance preferences [18], however wide range of outcomes were found also in children and adolescents in the presence of modulating factors such as eye contact, active approach or passive role, and whether an intervention (social interaction) was used [16, 19, 22]. First, our aim was to test whether interpersonal distance is greater in adult ASD than in control participants, as measured by the stop distance paradigm.

Social communication and interactions are tremendously complex processes that can be altered in autism at cognitive, behavioural and physiological levels. At the highest level, theory of mind difficulties can be observed in ASD [28, 29]. Making inferences about the mental state of another person requires more cognitive control during third-order mentalization. When arousal is high, more automated, self-centred thinking and behaviour take over [30]. We added attribution (mental state attributed to oneself or another person) as a modulating factor to capture this phenomenon during the interpersonal distance measurement. First, participants had to make a decision based on their own personal preference. Next, they were asked to estimate the comfortable distance for the experimenter.

The processing of facial expressions, particularly that of the eye region, is highly relevant in the regulation of social behaviour, including interpersonal distance. Facial emotion processing and emotion recognition is altered in autism [8, 31–34]. Constraining eye contact led to an exaggerated increase in amygdala activation, while decreased eye contact was associated with diminished amygdala response to faces in ASD [4, 35–37]. In addition, unconsciously avoiding eye contact results in further difficulties in reading socially important signals in ASD [38, 39]. These results suggest that altered amygdala functioning, including the regulation of eye contact, might have a substantial role in the disturbances of several aspects of social behaviour, such as personal proximity or interpersonal space regulation [27, 40, 41]. Therefore, in addition to the attribution, eye contact and no eye contact conditions were introduced to investigate the effect of these relevant factors in interpersonal distance regulation and in social communication.

Physiological response to sensory, social and emotional stimuli is suggested to be altered in ASD in general, however, the methodology used is highly variable and the results are inconsistent [42]. Since the classic electrophysiological experiment of Hutt et al. showed hyperarousal in children with ASD [43], the majority of studies that measured autonomic regulation (pupillometry, skin conductance, or cardiac measures) found atypical resting-state functions indicating either hyper- or hypoarousal in ASD according to a recent review [44]. Among healthy participants, Ferri et al. [45] found an association between respiratory sinus arrhythmia and interoceptive sensitivity (level of discomfort) in social situations. In a recent study Candini et al. found higher skin conductance response at closer distance, and it was even higher if the other person approached than when they moved farther away [46]. Another suitable tool to measure autonomic regulation is heart rate variability (HRV): heart rate is affected by both sympathetic and parasympathetic modulatory effects; thus, its variability might be a good marker of autonomic regulation, as higher HRV reflects parasympathetic activity [47]. Furthermore, a study found an association between HRV and cognitive flexibility in healthy individuals [48]. A recent meta-analysis showed that heart rate variability is reduced in ASD: baseline HRV and HRV reactivity during social stress were significantly lower in participants with ASD, but HRV reactivity performing cognitive tasks did not differ [49]. The reduced variability in the heart rate indicates an altered parasympathetic-sympathetic balance in ASD, suggesting the predominance of sympathetic activity and less flexible switching between autonomic states in ASD compared to neurotypicals. For these reasons, we measured interpersonal distance along with heart rate variability to examine their putative alterations and their relationship in ASD.

In this study, our main goal was to establish a comprehensive design to measure interpersonal distance and autonomic functions in ASD. Our first hypothesis was that in adult ASD we observe greater interpersonal distance. Second, we hypothesised that interpersonal distance is modulated by eye contact and attribution. Finally, we aimed to determine the role of autonomic functions in interpersonal distance regulation in ASD, expecting decreased baseline HRV and reduced HRV reactivity during the interpersonal distance task in ASD. It was also hypothesised that autonomic regulation, as characterised by HRV, could predict the preferred interpersonal distance in both study groups.

## Materials and methods

### Participants

In total, 45 adults participated in our research. Two control participants were excluded due to errors during data collection. The final sample consisted of forty-three participants, 22 were diagnosed with autism spectrum disorder (ASD) without intellectual disability or language impairment, and 21 were controls participants without autism (CP). The two groups did not differ in age, gender and education (Table 1). All participants with ASD were diagnosed by trained clinicians, the diagnoses were confirmed with Autism Diagnostic Interview-Revised (ADI-R) and Autism Diagnostic Observation Schedule, IV-module (ADOS-IV.) [50, 51]. Twelve participants had one or more comorbid disorders (attention deficit hyperactivity disorder (5), obsessive-compulsive disorder (3), generalised anxiety disorder (2), bipolar disorder (1), depression (1), and schizophrenia (1)). Participants with ASD were recruited from the outpatient unit of the Department of Psychiatry and Psychotherapy, Semmelweis University. Control participants were recruited by advertisement. Exclusion criteria were history of psychiatric or neurological illness, developmental anomalies and any first degree relatives with ASD diagnosis.

**Table 1:** Demographics and clinical characteristics.

		ASD (*N* = 22)		CP (*N* = 21)		Statistic	*p*	*effect size*	
		*N* (M/F)		*N* (M/F)		*χ* ^*2*^	*p*		
**Gender**		18/4		14/7		1.296	0.255		
		*Min-Max (Mean)*	*SD*	*Min-Max (Mean)*	*SD*	*Mann-Whitney U*	*p*	*r*	*95% CI* *lower*, *upper*
**Age**	(years)	19–44 (27.59)	7.25	19–43 (25.86)	6.44	191.00	0.336	0.173	-0.173, 0.481
		*Mean*		*Mean*					
**Education**	(years)	15.64	3.69	16.00	3.33	248.50	0.677	-0.076	-0.401, 0.267
**AQ**		31.09	6.63	15.05	5.63	18.50	<0.001	0.920	0.845, 0.959
**MZQ**		51.68	9.53	38.29	9.30	71.50	<0.001	0.690	0.462, 0.833
**AAS**	anxious	22.64	5.77	16.81	6.06	114.50	0.005	0.504	0.203, 0.718
	avoidant	41.23	8.80	31.71	8.12	93.50	<0.001	0.595	0.324, 0.776
**ASRS**	Part A	13.23	4.02	9.52	3.84	110.50	0.003	0.522	0.225, 0.730
	Part B	26.64	9.19	16.62	5.83	88.00	<0.001	0.619	0.358, 0.790
**STAI-T**		55.46	11.63	44.29	9.09	105.50	0.002	0.543	0.254, 0.743
**ADOS-IV.**		9.96	3.35	-	-	-	-	-	-
**ADI-R**		34.82	7.74	-	-	-	-	-	-
		***N*** (Y/N/n.a.)		***N*** (Y/N/n.a.)		*χ* ^*2*^	*p*		
**Caffeine**	regular	18/2/2		15/5/1		1.558	0.212		
	within 12h	13/7/2		13/6/2		0.051	0.821		
**Smoking**		2/18/2		4/16/1		0.784	0.376		
**Exercise**		18/4/0		17/3/1		0.076	0.782		

ASD: Autism Spectrum Disorder, CP: Control Participant, N: sample size, SD: standard deviation, r: rank biserial correlation, 95% CI: 95% confidence interval, M: male, F: female, Y: yes, N: no, n.a.: not available, AQ: Autism-Spectrum Quotient, MZQ: Mentalization Questionnaire, AAS: Adult Attachment Scale, ASRS: Adult ADHD Self-Report Scale, STAI-T: State-Trait Anxiety Inventory—Trait, ADOS-IV: Autism Diagnostic Observation Schedule—IV. module, ADI-R: Autism Diagnostic Interview-Revised

Participants (and legal guardians if applicable) provided written informed consent and did not receive financial compensation for their attendance. The study was conducted in accordance with the Declaration of Helsinki, and it was approved by the Regional and Institutional Committee of Science and Research Ethics, Semmelweis University, Budapest, Hungary (SERKEB No.: 145/2019) from October 2019 to February 2020. The experiment took place at the Laboratory of Brain, Memory and Language Lab, Eötvös Loránd University, Budapest.

### Experimental paradigm—Interpersonal distance task

**Fig 1:**
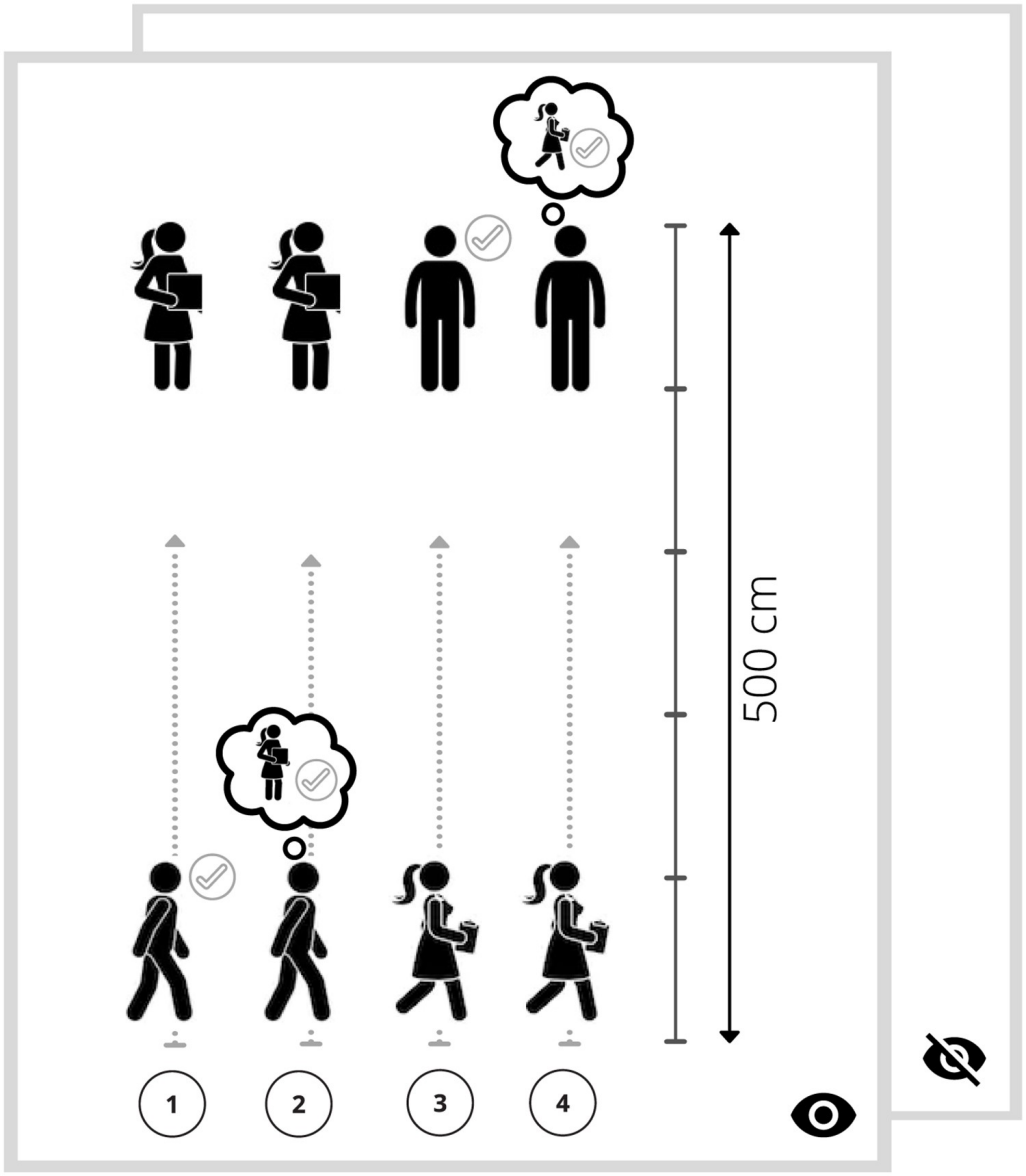
Experimental setting. The modified version of the stop distance paradigm. First, (1) participants were approaching actively and were asked to stop where it still felt comfortable for them. Next (2) participants were approaching actively and were asked to stop where they thought it was still comfortable for the experimenter. Then (3) participants stood passively and were asked to stop the experimenter where it was still comfortable for them; finally (4) participants stood passively and were asked to stop the experimenter where they thought it was still comfortable for the experimenter. Participants repeated this procedure twice with and without eye contact; the order of the latter two conditions was randomised across participants.

In our study, we measured social distance regulation. Participants underwent an interpersonal distance measurement, a modified version of the stop-distance paradigm [27]. In all conditions, the participant and the experimenter started from the opposite endpoints of the tape measure (five metres) stuck to the floor. They were asked to consciously focus on keeping a comfortable social distance, eight times in total, in the following order. First, (1) participants were approaching actively and were asked to stop where they still felt comfortable. Next, (2) participants were approaching actively and were asked to stop where they thought it was still comfortable for the experimenter. Then (3) participants stood passively and were asked to stop the experimenter where it was still comfortable for them; finally, (4) participants stood passively and were asked to stop the experimenter where they thought it was still comfortable for the experimenter. Participants repeated this procedure twice, with and without eye contact: either the experimenter was looking at the participant (eye contact condition) or the papers she was holding (no eye contact condition). The order of these two conditions was randomised across participants (Fig 1). During the statistical analysis active and passive conditions were pooled (averaged) together.

### Heart rate monitor

A wearable Polar H10 device was placed on participants’ chests, which recorded heart rate (HR) during the whole experiment. We measured cardiac interbeat intervals (RR intervals) using Polar H10 heart rate monitor chest strap (Polar Electro Oy, Kempele, Finland) [52], which is a valid device to measure RR interval signals [53]. The HR monitor was connected to a Samsung Galaxy Tablet via Bluetooth. We used the Elite HRV application to export the recorded RR intervals as.txt files. We measured heart rate variability (HRV) under two different conditions for a duration of 60 seconds: 1) at baseline and 2) during the intentional interpersonal distance task (1 minute after starting the distance task, to avoid measuring the mild physical activity related artifacts that may have resulted from reaching a new postural position) using the Root Mean Square of Successive RR interval Differences (RMSSD) method [54]. Additionally, we calculated RMSSD at the preceding ten-second time window of trigger points set by researchers. These triggers corresponded to the time when participants arrived at their final location of each condition.

### Distance measuring: Obimon Prox

In order to synchronise the distance data with the HRV data, both the experimenter and the participant wore a distance measuring device. The Obimon Prox [55] measures the distance and the relative orientation between two wearable devices in real-time. The devices use Ultra Wide Band (UWB) technology and the Symmetrical Double-Sided Two-Way Ranging (SDS-TWR) method [56] to both determine the distance between each other by emitting very short and low power radio transmissions and measure the so-called time-of-flight (ToF) with very high precision between transmission and reception. The resolution of the measurement is in the range of a few centimetres, while the absolute precision is approximately 10 centimetres. The relative orientation is defined as the difference between the angles between the two devices taking the Earth’s magnetic field as a reference. For increased precision, the device uses sensor fusion involving magnetometer, accelerometer, and gyroscope sensors. The results of the measurements are collected over Bluetooth LE wireless technology to a laptop computer and evaluated in real-time.

### Procedure

Participants wore a Polar H10 and an Obimon Prox device during the whole experiment. They placed the wearable device on themselves before the experiment started, then waited five minutes while calibrating and registering 60 seconds of resting heart rate and HRV.

Next, participants completed the interpersonal distance task. Then, after a short break, they completed a computerised neurocognitive test battery—measuring working memory, executive functions, attention, inhibition, implicit learning, faux pas. These results are not reported in this paper. Finally, they completed computerised versions of self-report questionnaires (AQ: Autism-Spectrum Quotient, MZQ: Mentalization Questionnaire, AAS: Adult Attachment Scale, ASRS: Adult ADHD Self-Report Scale, STAI-T: State-Trait Anxiety Inventory—Trait; see Table 1 and S1 File).

To avoid the sensory over-reactivity effect, experimenters did not wear neither any jewellery nor perfume and had been asked to refrain from eating spicy food before the experiment. They wore simple, casual, non-coloured clothes (jeans and black T-shirt). The room was curtained and evenly lit artificially.

### Data preprocessing and analysis

Preparation of HRV data was carried out using Python 3.7 with NumPy 1.20.1 [57], pandas 1.2.3 [58], and SciPy 1.6.1 [59] data processing packages. Since the samples were measured at a different rate for the Polar H10 (one sample per second) and the Obimon Prox (one sample per milliseconds) devices, we resampled the Obimon data by taking the median for each second. Missing data were dropped from the analysis (9.52% of the control group and 18.2% of the ASD group did not have a complete HRV record). To synchronise HRV with the proximity data we needed to obtain the timestamps for each file containing the RR intervals. The first timestamp was obtained from the name of the file which indicated the start time of the recording. Since the exported files only contained the RR intervals without a timestamp for each sample, the interval values themselves were used to create the time elapsed since the first sample. As RR intervals annotate the time between two successive heartbeats, it was possible to append the value of the RR interval to the time of the previous sample. After obtaining the timestamps, data points were replaced with the median if they indicated RR of 1200 milliseconds (ms) or above, or if their absolute Z score was higher than 2. Triggers added to the distance data (see Distance measuring) were adjusted manually if needed. HRV was estimated as the root mean square of successive RR interval differences (Root Mean Square of Successive Differences, RMSSD) since this measurement is relatively resistant to by-products caused by breathing [60], and can be obtained for a shorter (10 seconds) period of time [61]. Calculations were done by the following formula (1):

$$RMSSD=\sqrt{\frac{1}{N-1}\sum_{i=0}^{n}{\left(RR_{i+1}-RR_{i}\right)}^{2}}$$ (1)

Baseline HR and HRV were measured and calculated for 60 seconds (s) at baseline, and reactive HR and HRV were measured during the interpersonal distance task one minute after starting the explicit paradigm (from +60 s to +120 s).

Furthermore, we calculated RMSSD around time points where interpersonal distance data were reported. To calculate RMSSD for each explicit condition, eight local minimums of the distance data were determined from data recorded by Obimon Prox. These eight time points indicate the shortest distances between the participant and the experimenter, corresponding to the time point when the reported distance was reached. RMSSD was calculated for an interval starting 10 seconds prior to reaching the reported distance.

### Statistical analysis

Statistical analysis was accomplished using R Version 3.6.3 [62], RStudio Version 1.2.1335 [63], and JASP Version 0.14.0.0 and 0.16.4.0 [64]. First, to measure if the two study groups do differ regarding age, gender, education, caffeine intake, smoking, exercise and scores on questionnaires, we conducted nonparametric Mann-Whitney U (Wilcoxon rank-sum) tests and a Chi-square test. To measure the effect of different conditions and study groups on the distance and HRV data, mixed-design ANOVA tests were applied, while in the case of significant interaction effects, post hoc tests with Bonferroni correction were used. We performed Bayesian ANOVAs as well, which enabled us to detect null effects. Using its default prior, we calculated Bayes Factor_exclusion_ (BF_excl_) values in JASP 0.16.4.0. We compared the models to the null model (which included the subject variable and random slopes) in each case, and we calculated the BF_excl_ values across all models. BF_excls_ reflect how much more likely it is that the effect does not exist (H_0_) compared to that it does (H_1_), given the data. The BF_excl_ values above 1 support the exclusion of the given factor from the model, while values below 1 support the inclusion. Values close to one indicate that there is not enough evidence to support neither inclusion nor exclusion. Furthermore, for the sake of transparency, we reported BF_01_ values and errors (%) in S4 Table. As there was already a baseline difference in HRV between the two groups, the HRV values were standardised in the interpersonal conditions for further comparisons. Associations between distance, HRV, and scores of psychometric questionnaires and diagnostic tests were analysed with Spearman’s rank-order correlations. Analyses were performed, and visualisations were created with R-packages *dplyr* [65], *ggplot2* [66], *psych* [67], *gridExtra* [68], *ggpubr* [69], *readxl* [70], *corrplot* [71], *Hmisc* [72], *varian* [73].

## Results

### Is preferred interpersonal distance different in ASD?

**Fig 2:**
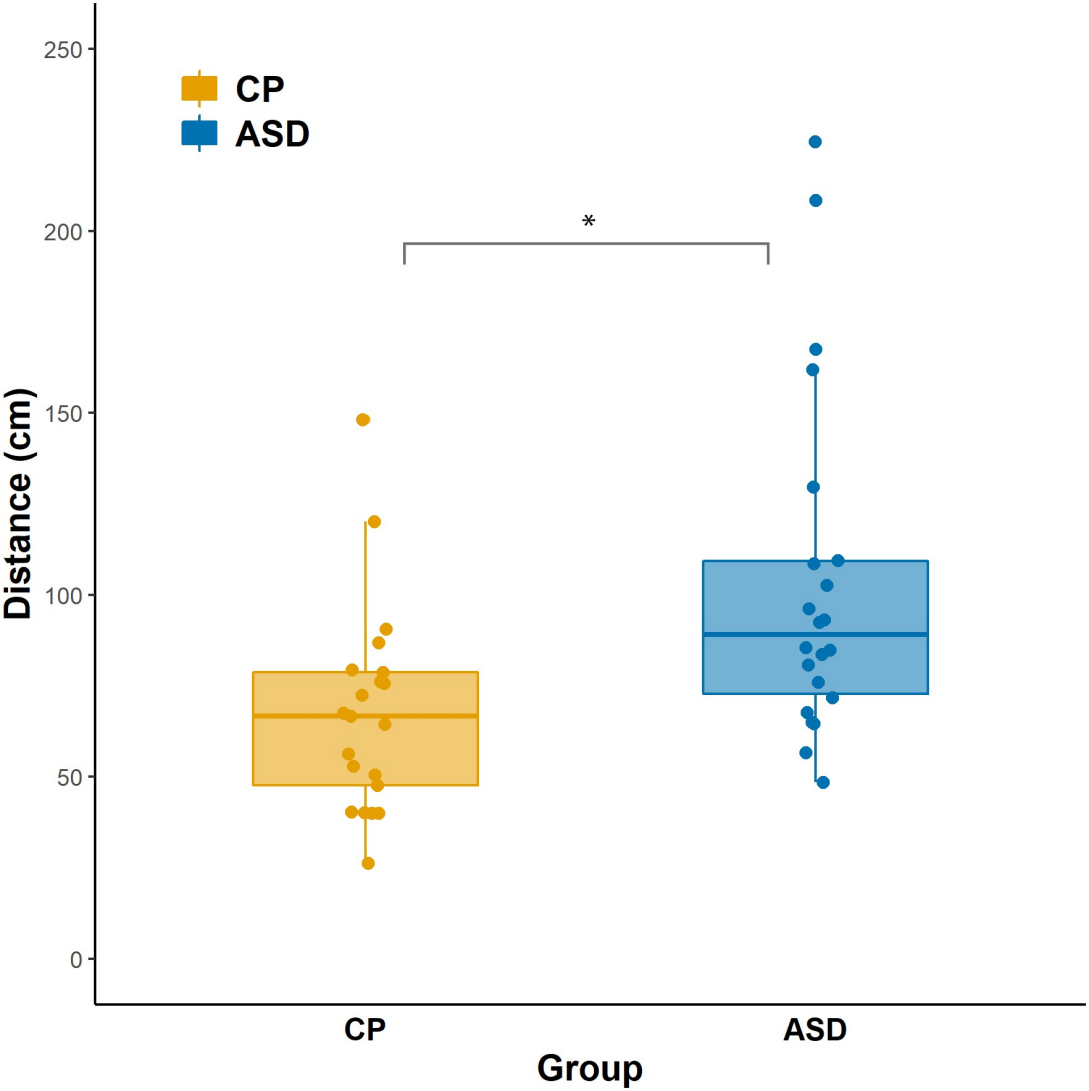
Interpersonal distance in cm. Dots represent the mean of distance data of eight conditions for each individual. The top and the bottom of the box show the upper (Q3) and lower (Q1) quartiles, the line dividing the box represents the median, and notches show a 95% confidence interval around the median. Asterisks indicate significant group differences. Orange: control participants, blue: participants with ASD.

To test if the interpersonal distance was different, or if eye contact and attributions had different modulatory effects in the two study groups, we used two-way mixed-design ANOVA on the interpersonal distance as a dependent variable, where the between-subject factor was the Group (ASD/CP), within-subject factors Eye contact (Yes/No) and Attribution (Self/Other). The Group main effect (*F*(1,41) = 8.999, *p* = .005, *η*^*2*^_*p*_ = 0.180) was significant, participants with ASD preferred larger distances in general (*M*_*(ASD)*_ = 103.670, *SD*_*(ASD)*_ *=* 47.322; *M*_*(CP)*_ *=* 67.690, *SD*_*(CP)*_ *=* 28.589; mean difference = 35.980, 95% CI [11.757, 60.203]) (Fig 2). For Bayesian analyses, see Table 2. According to the post hoc power analysis, the group difference was detected with 98% power. Levene’s test showed that the variances were equal. For the descriptive statistics of all conditions see S1 Table.

**Table 2:** Bayesian ANOVA: Effects.

**Interpersonal distance**			
**Effects**	**P(excl)**	**P(excl|data)**	**BF**_**excl**_
Group	0.26	0.08	0.24
Eye Contact × Group	0.68	0.75	1.38
Attribution × Group	0.68	0.81	1.97
Eye Contact × Attribution × Group	0.95	0.99	7.82
**HR baseline vs experiment**			
**Effects**	**P(excl)**	**P(excl|data)**	**BF**_**excl**_
Group	0.40	0.48	1.36
Time × Group	0.80	0.86	1.53
**HRV baseline vs experiment**			
**Effects**	**P(excl)**	**P(excl|data)**	**BF**_**excl**_
Group	0.40	0.21	0.40
Time × Group	0.80	0.49	0.24
**HRV in interpersonal context**			
**Effects**	**P(excl)**	**P(excl|data)**	**BF**_**excl**_
Group	0.26	0.65	5.17
Eye contact × Group	0.68	0.95	9.31
Attribution × Group	0.68	0.96	9.99
Eye contact × Attribution × Group	0.95	1.00	146.00

*Notes*. The Effects column shows the main effects and interactions. The P(excl) column indicates the prior exclusion probability, and the P(excl|data) denotes the posterior inclusion probability. The BF_excl_ column shows the exclusion Bayes Factors. BF_excl_ values below 1 support the inclusion and values above 1 the exclusion of the given factor.

#### Does eye contact or attribution affect interpersonal distance?

We performed the above-described two-way mixed-design ANOVA, and the main effect of eye contact, or attribution resulted in the following. Eye-contact (*M*_eye_(*SD*) = 88.756 (47.616), *M*_no_eye_(*SD*) = 83.442 (40.116), *F*(1,41)_eye_cont_ = 3.005, *p* = .091, *η* ^*2*^_*p*_ = 0.068, 95% CI [-0.859, 11.267]) showed a trend, and attribution (*M*_self_(*SD*) = 84.913 (53.165), *M*_other_(*SD*) = 87.285 (37.378), *F*(1,41)_attrib_ = 0.248, *p* = .621, *η* ^*2*^_*p*_ = 0.006, 95% CI [-12.691, 7.671]) did not have a significant main effect, indicating that participants attributed similar comfortable personal distance to the experimenter as to themselves (Fig 4a and 4b). The Eye contact × Attribution interaction resulted in a trend (*F*(1,41)_eye×attrib_ = 3.011, *p* = .090, *η* ^*2*^_*p*_ = 0.068; the Group × Eye contact, Group × Attribution, Group × Eye contact × Attribution interactions were not significant ((*F*(1,41)_group×eye_ = 2.480, *p* = .123, *η* ^*2*^_*p*_ = 0.057, (*F*(1,41)_group×attrib_ = 1.378, *p* = .247, *η* ^*2*^_*p*_ = 0.033, (*F*(1,41)_group×eye×attrib_ = 0.016, *p* = .900, *η* ^*2*^_*p*_ < 0.001)). For Bayesian analyses, see Table 2.

### Are heart rate and heart rate variability altered in ASD?

**Fig 3:**
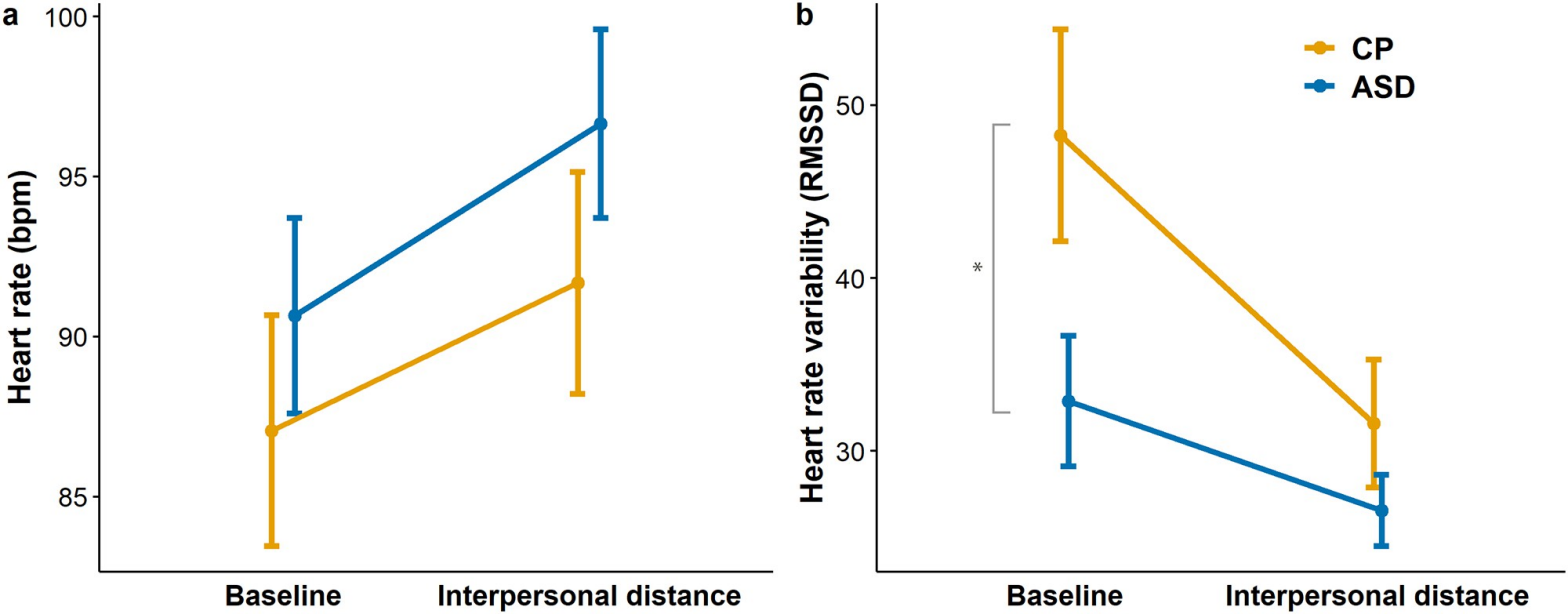
Heart rate and heart rate variability. Panel A: Baseline and reactive (interpersonal conditions) heart rate in beat per minute (bpm). Panel B: Baseline and reactive (interpersonal conditions) heart rate variability (RMSSD). Error bars: standard error of the mean. Asterix indicates significant group difference. Orange line: neurotypical participants, blue line: participants with ASD.

Measuring the heart rate, heart rate variability, and the influence of interpersonal condition on them, we used mixed-design ANCOVA, with the between-subject variable of Group (ASD/CP), and within-subject variable Time (baseline/interpersonal) on HR and HRV as dependent variables respectively. In general, participants with ASD had a slightly higher heart rate (*M*_*baseline*_ *=* 90.65, *SD* = 12.95; *M*_*interpersonal*_ = 96.66, *SD* = 12.49) than CP participants (*M*_*baseline*_ *=* 87.06, *SD* = 15.74; *M*_*interpersonal*_ = 91.68, *SD* = 15.08) (Fig 3), however, the Group main effect was not statistically significant (*F*(1,35) = 0.875, *p* = .356, *η* ^*2*^_*p*_ = 0.024). The main effect of Time was significant (*F*(1,35) = 38.068, *p* < .001, *η* ^*2*^_*p*_ = 0.521), but the Group × Time interaction was not (*F*(1,35) = 0.647, *p* = .427, *η* ^*2*^_*p*_ = 0.018). As caffeine intake, sport and smoking could influence the heart rate [74–76], we included them as covariates, but it did not change the results on the Group main effect (*F*(1,27) = 1.489, *p* = .233,^*2*^_*p*_ = 0.052). It means, that in both groups we measured the highest HR during the intentional interpersonal distance task, and it was significantly higher than baseline (*t*_ASD_ = 5.866, *p* < .001, 95% CI [2.554, 9.454], *t*_CP_ = 3.846, *p* = .003, 95% CI [1.260, 7.977]) according to the post hoc tests, where *p*-values were adjusted by using Bonferroni-correction. Levene’s test showed that the variances were equal (Fig 3a).

Heart rate variability (HRV) was higher in the CP group (*M*_*baseline*_ *=* 48.26, *SD* = 26.68; *M*_*interpersonal*_ = 31.60, *SD* = 16.11) than in ASD (*M*_*baseline*_ *=* 32.90, *SD* = 15.97; *M*_*interpersonal*_ = 26.57, *SD* = 8.79). Again, measuring the different effects of the conditions we used mixed-design ANCOVA, with the same within- and between-subject variables described above. The group main effect (*F*(1,35) = 3.470, *p* = .071, *η* ^*2*^_*p*_ = 0.090) showed a trend. The Time main effect (*F*(1,35) = 22.744, *p* < .001, *η* ^*2*^_*p*_ = 0.394) and Group × Time interaction (*F*(1,35) = 4.598, *p* = .039, *η* ^*2*^_*p*_ = 0.116) were significant. The post hoc test showed significant difference between the baseline and interpersonal condition (*t* = 4.769, *p* < .001, 95% CI [6.600, 16.383]), but this difference originates from the significant difference within the CP group (*t*_CP_ = 4.956, *p* < .001, 95% CI [7.258, 26.059]), whereas HRV did not differ significantly in ASD group between the two conditions (*t*_ASD_ = 1.831, *p* = .276, 95% CI [-3.333, 15.982]) (Fig 3b). For Bayesian analyses of the HR and HRV differences, see Table 2. The more pronounced difference between baseline HRV and HRV during the interpersonal task suggests a greater autonomic regulation capacity of CPs, whereas in ASD participants, the baseline HRV was already low, preventing further decrease and raising the possibility of a floor effect. There was no significant difference in HR or HRV in any time condition between subgroups of participants with ASD with and without comorbidities.

#### Does eye contact or attribution affect interpersonal heart rate variability?

We used two-way mixed-design ANOVA on heart rate variability as dependent variable, where the between-subject factor was the Group (ASD/CP), within-subject factors Eye contact (Yes/No) and Attribution (Self/Other). As the baseline HRV was higher in the CP group, we used standardised HRV to the given person’s baseline HRV here. HRV during the interpersonal distance task (measured before the time point reported distance was reached) was numerically higher in the CP group, but the difference was not significant between groups (Group main effect *F*(1,32) = 0.0002, *p* = .988, *η*^*2*^_*p*_ < 0.001). For descriptive statistics see S2 Table.

**Fig 4:**
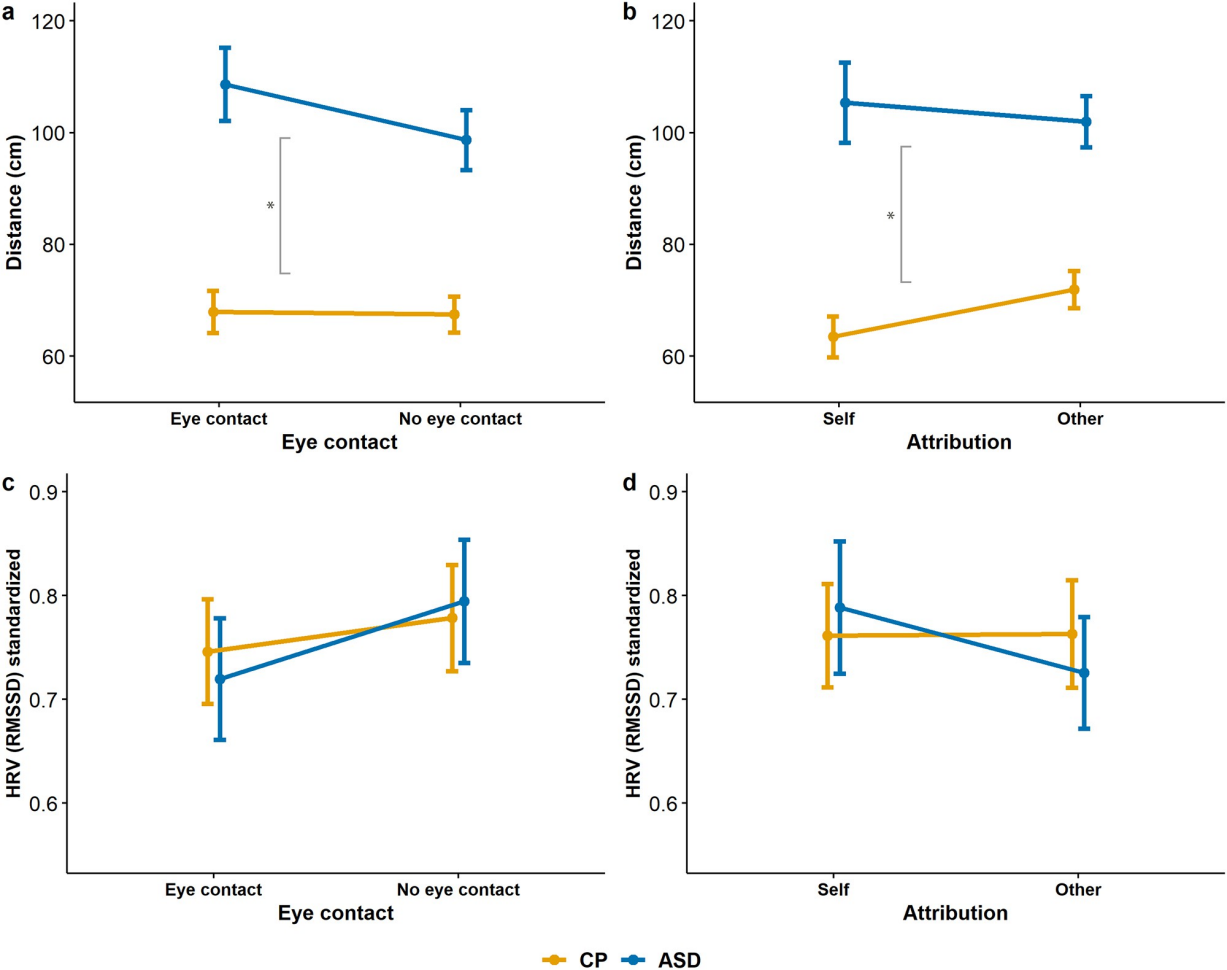
Interpersonal distance and HRV differences in different conditions. Panel a-b Interpersonal distance in cm. Panel a: With or without eye contact, Panel b: Attribution to self or the other. Panel c-d Heart rate variability in explicit conditions at a reported distance. Panel c: With or without eye contact, Panel d: Attribution to self or the other. Error bars: standard error of the mean. Asterisks indicate significant group differences. Orange line: control participants, blue line: participants with ASD.

Neither the main effect of eye contact (*F*(1,32) = 2.209, *p* = .147, *η* ^*2*^_*p*_ = 0.065) or attribution (*F*(1,31) = 0.328, *p* = .571, *η* ^*2*^_*p*_ = 0.010) nor their interaction with each other (*F*(1,32) = 0.117, *p* = .735, *η* ^*2*^_*p*_ = 0.004) or group (eye contact × group: *F*(1,32) = 0.817, *p* = .373, *η* ^*2*^_*p*_ = 0.025; attribution × group: *F*(1,32) = 0.554, *p* = .462, *η* ^*2*^_*p*_ = 0.017; eye contact × attribution × group: *F*(1,32) = 0.520, *p* = 0.476, *η*^*2*^_*p*_ = 0.016) were significant (Fig 4c and 4d, BF_excl_s are shown in Table 2). Autonomic functioning might be influenced by smoking, exercise, regular caffeine consumption, or the actual caffeine intake before the experiment. There was no difference between groups (see Table 1), however, including these variables as covariates did not change the results.

The interpersonal distance (Panels a-b) and heart rate variability data measured by the RMSSD method (Panels c-d) are presented in Fig 4 to introduce their characteristics in eye contact (Yes/No) and attribution (Self/Other) conditions in the two study groups.

#### Is there any correlation between HRV, distance, and psychometric data? Exploratory analysis

**Fig 5:**
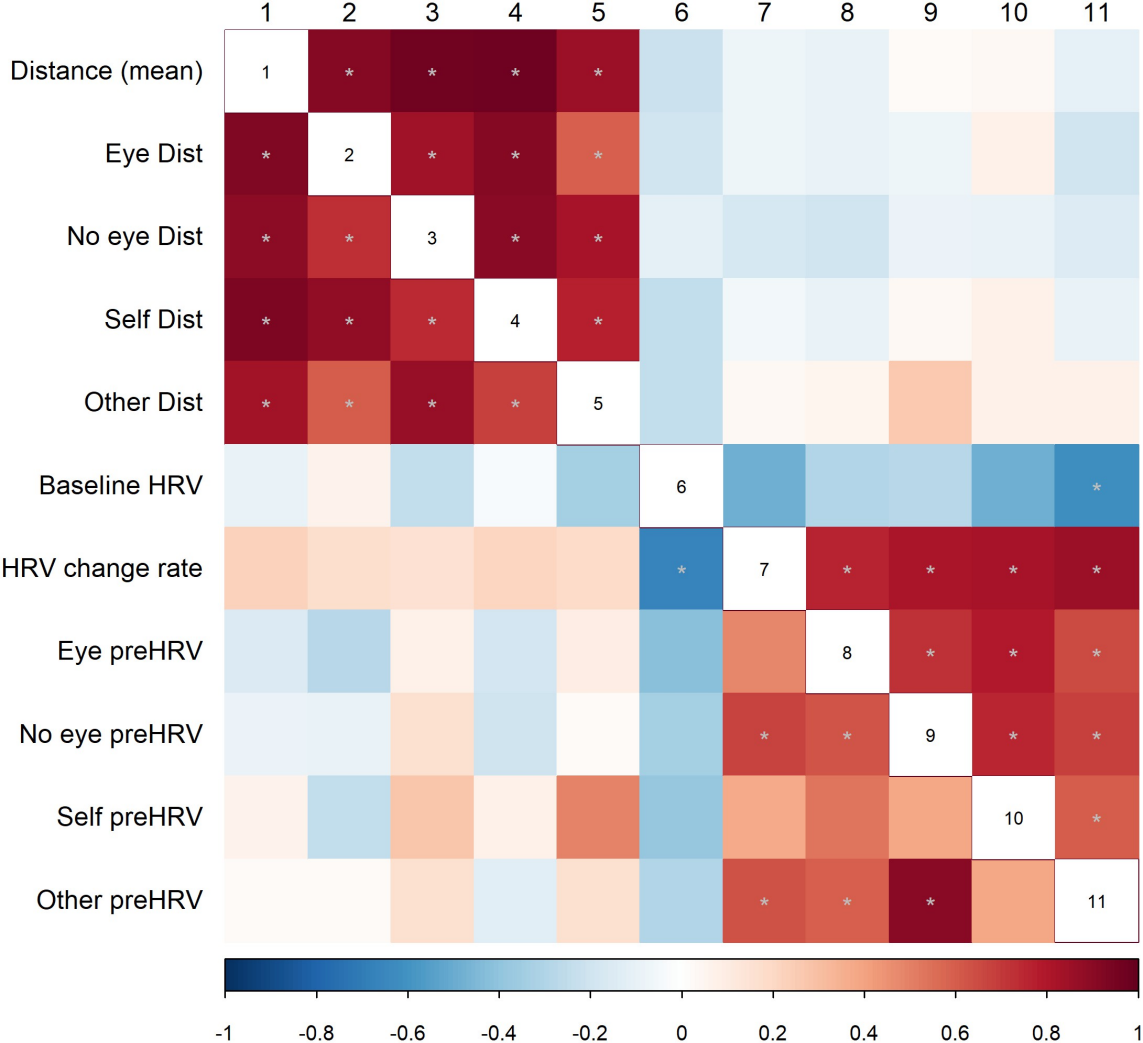
Correlations between interpersonal distance and heart rate variability at the baseline and during the intentional interpersonal distance conditions. Dist = distance, HRV = heart rate variability, preHRV = 10s RMSSD, Eye = eye contact, No eye = no eye contact, Active = active moving, Passive = standing, Self = attribution to self, Other = attribution to the other conditions. Upper triangle: control participants, lower triangle: participants with ASD. Warm colours refer to positive, cold colours refer to negative Spearman rank correlation *rho* values, grey asterisk marks the significant *p* values after (fdr) correcting for multiple comparisons.

**Fig 6:**
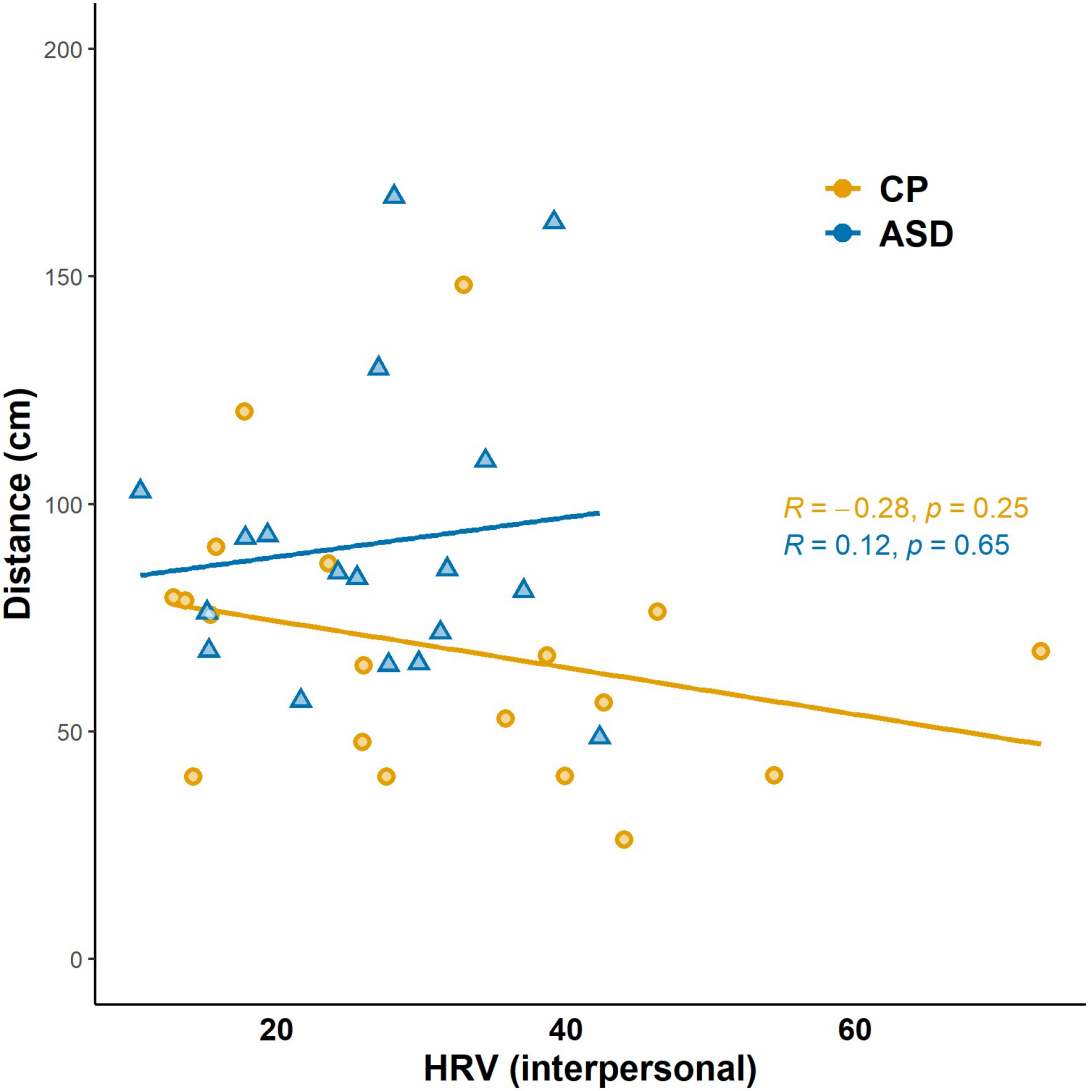
Correlation between mean distance (in cms) and HRV (60 s) during interpersonal distance task in the two groups. Orange line: neurotypical participants, blue line: participants with ASD.

Correlation analysis was highly exploratory, due to the small sample size, but it might be suitable for further hypothesis generation. Interpersonal distance and HRV data are characteristic of an individual, and the examined modulatory factors have little or no effect on them, neither in the CP (Fig 5, upper triangle) and the ASD samples (Fig 5, lower triangle). FDR (false discovery rate; Benjamini–Hochberg procedure) method was conducted correcting for multiple comparisons. The correlation between the mean interpersonal distance and HRV during the interpersonal distance task was not significant, however, it tends to point in different directions in the two groups (Fig 6).

To test whether HRV during the experiment predicted the preferred interpersonal distance, and whether autism moderates this relationship, we conducted a linear regression analysis with the dependent variable of interpersonal distance and the predictors standardised HRV and group (ASD/CP) and their interaction. The criteria of lack of multicollinearity (*VIF* = 1), autocorrelation (*Durbin-Watson* = 1.912) and heteroscedasticity were met, however, the residuals violated the normal distribution, thus, we used bootstrapping (10000 iterations) to estimate the unstandardized coefficients. The model was significant [*F*(3,138) = 4.210, *p* = .007], it explained 8.4% of the variance of the interpersonal distance. The group was a significant predictor of the interpersonal distance (*B*_*bootstrap*_ = -24.905, *SE* = 12.973, *p* = 0.042), reflecting the same difference we found with the ANOVA above. However, the HRV and the HRV × group interaction were nonsignificant predictors (*B*_*bootstrap*_ = -4.737, *SE* = 11.661, *p* = 0.811; *B*_*bootstrap*_ = 3.635, *SE* = 15.208, *p* = 0.811, respectively), meaning that HRVs did not predict the interpersonal distance, and ASD did not moderate this relationship either.

**Fig 7:**
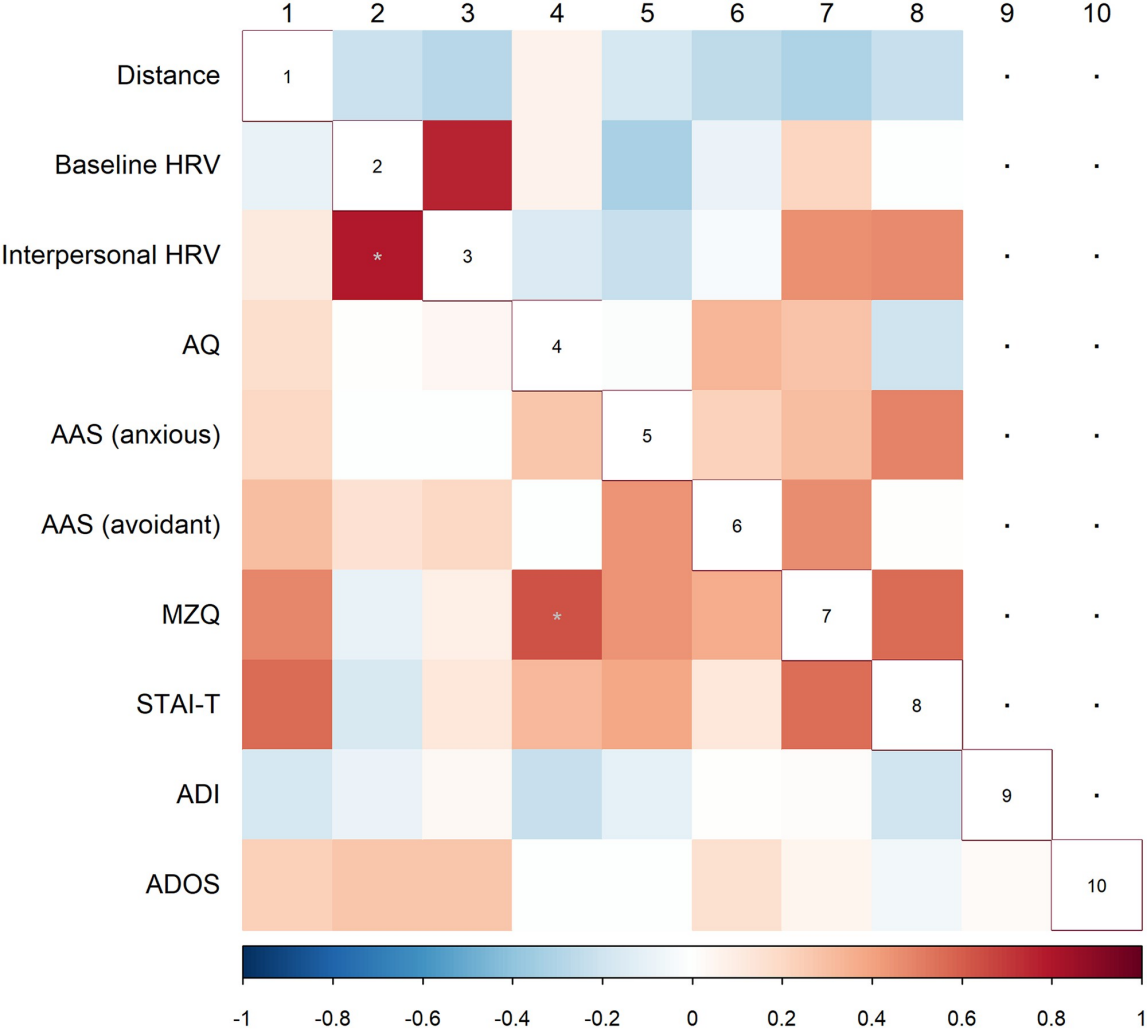
Correlations between interpersonal distance, heart rate variability at the baseline and during the interpersonal distance conditions, and psychometric data. HRV = heart rate variability, AQ = Autism-spectrum Quotient, AAS = Adult Attachment Scale, MZQ = Mentalization Questionnaire, STAI-T = State-Trait Anxiety Inventory, Trait, ADI = Autism Diagnostic Interview-Revised, ADOS = Autism Diagnostic Observation Schedule. Upper triangle: neurotypical participants, lower triangle: participants with ASD. Warm colours refer to positive, cold colours refer to negative Spearman rank correlation *rho* values, grey asterisk marks the significant *p* values after (fdr) correcting for multiple comparisons.

Participants also completed self-report questionnaires. The results shown here are highly exploratory given the low number of participants and the limitations of the validity of psychological questionnaires. Results of psychometric questionnaires showed weak or no association with distance and HRV results (Fig 7); however, the association with psychometric questionnaires in ASD showed a different pattern than in CP. High trait anxiety level, poor mentalization, and attachment were weakly associated with greater interpersonal distance ASD (Fig 7, lower triangle, first column), but neither of these correlations remained significant after FDR correction for multiple comparisons, only HRV at baseline and during the interpersonal condition, AQ and mentalization scores were correlated in ASD group.

## Discussion

Our study aimed to investigate interpersonal distance regulation and the underlying autonomic response regulation in autism spectrum disorder. To this end, we introduced a paradigm combining interpersonal distance measurement and physiological parameter registration in an interpersonal experimental setting in groups of adult participants with ASD and their matched neurotypical controls. We found increased interpersonal distance, decreased baseline heart rate variability and decreased HRV reactivity in ASD, indicating lower parasympathetic activity in ASD. The difference was expected to be more pronounced when the experimenter maintained eye contact and participants were requested to determine their own comfortable distance during the interpersonal distance task. Still, the modulatory effect of these factors was not significant.

The interpersonal distance was measured using a modified version of the stop distance paradigm to assess how far participants prefer to stand from another person and whether there is a difference between ASD and CP group in this respect. Participants were directly instructed to define a still comfortable distance from the experimenter. Usually, in a stop distance paradigm, the participant and the experimenter are facing each other at the endpoints of a 300 to 600 cm long line along which the participants set their preferred interpersonal distance. In our experiment, we have chosen 500 cm as the initial distance, which includes all four distance zones of interpersonal space (intimate, personal, social and public) according to Hall’s proxemic rules [77]. During this task, participants set distances on average within the personal space (far zone ~75–120 cm, close zone ~45–75 cm, the zones between the intimate distance and personal distance). However, as expected, participants with ASD set significantly greater distances than CPs: around the far end of the personal space, or even farther. The social space (the zone between personal and social distance 120–370 cm) is reserved for strangers or new acquaintances [77]. We can speculate that this difference may affect the non-verbal message conveyed by a specific distance in real-life interpersonal situations: neurotypicals may perceive their ASD peers as withdrawn, distant, whereas for people with ASD the occupied space is at a comfortable distance reserved for a familiar; conversely, people with ASD may perceive interpersonal distance, implicitly considered to be average by others, as too close, even intrusive.

Prior to our experiment, studies examined interpersonal space regulation in ASD suggesting that interpersonal space regulation is altered in ASD in childhood, but the nature and direction of the disturbance are not entirely consistent. Autistic children preferred significantly larger interpersonal distance than neurotypical control participants [16, 22]. A study examining adolescents with ASD also concluded that their space regulation was altered. Interestingly, this conclusion was derived from opposing results: adolescents with ASD preferred shorter interpersonal distance than neurotypical controls [19]. Although in this study from Japan, neurotypical participants preferred longer personal distances (ca. 130–150 cm depending on condition) than in other cohorts, but the distances preferred by ASD participants were comparable to our and Kennedy and Adolphs’s results. The only adult study observed no differences between participants with ASD and neurotypical controls [18] (ca. 70–100 cm). This raises the possibility of implicitly learned external cues, cultural differences in social rules and customs (e.g. western versus eastern cultures), including personal space arrangement that affect neurotypicals more than people with ASD. Gender also might have an impact on interpersonal distance preferences. In this study, the experimenter was female regardless of the gender of the participants, who were predominantly males. We decided to choose female experimenters due to the overrepresentation of female professionals in therapeutic, educational, and most care-providing settings. Although ASD is more prevalent among males, and the gender ratio of our small sample is also in line with that observed in the population, we could not exclude that the difference we found had been influenced by this experimental arrangement. However, the distribution of gender in the two groups did not differ significantly, reducing the chance that gender itself affects the results. Our results suggested that adult individuals (a homogenous white Caucasian, Central European, mostly male sample) with ASD prefer greater interpersonal distance (from female experimenters) than their neurotypical controls.

Several factors can influence the interpersonal distance between the experimenter and the participants [13, 19, 22]. Eye contact has been previously shown to affect the preferred interpersonal distance of ASD and neurotypical adolescents: in eye contact conditions when participants held passive roles, they preferred larger interpersonal distance, regardless of which study group they belonged to, and this effect did not emerge when holding active roles [19]. In our study we failed to find any significant modulatory effect of the eye contact, in contrast with the “eye avoidance” hypothesis [36, 37]. Reciprocal social interactions are impaired in ASD, leading to a weaker adaptation to another person’s perspective. Therefore, we also introduced a condition that requires higher-order mentalization, but surprisingly our results did not confirm a significant modulatory effect of attribution either. These results might suggest that participants with ASD are capable of modifying their behaviour according to others’ aspects to a similar extent as neurotypical controls, in contrast with previous results [78, 79]. We can speculate from the results that eye contact and attribution at this simple level (setting a comfortable interpersonal distance) do not have a significant effect, but rather become relevant in the process of communication, during more complex reciprocal social interactions. Despite the two groups having different means of social distance or heart rate variability, the interpersonal processes appeared to be similar on this level.

An invention in our experimental design is that we combined interpersonal distance measurement with heart rate registration. In social behaviour parasympathetic regulation, the flexibility of vagal tone plays an important role according to Porges’ polyvagal theory [80–82]. Higher resting HRV was found to be associated with cooperative behaviour, using less disengagement and more socially adaptive emotion regulation strategies among healthy adults [83, 84]. Variables influenced by parasympathetic regulation (e.g., respiratory sinus arrhythmia) are related to emotion recognition and symptom severity in ASD [85]. In line with the results of previous studies corroborating altered autonomic nervous system functioning in ASD [44, 49, 86], we found reduced baseline heart rate variability in participants with ASD spectrum disorder. The average heart rate (reflecting sympathetic activity) was slightly higher in the ASD group than in neurotypicals, but this difference was not significant. In social situations, not just the baseline but also the regulatory capacity must be taken into account, skin conductance is elevated at closer distances among healthy participants [46]. Previous studies showed that the HRV decrease induced by participating in a social situation was lower, assuming a decreased regulatory capacity in the ASD group [49]. We used the RMSSD method to measure HRV in order to capture the parasympathetic regulation rather than sympathetic arousal [54]. We found a significant HRV decrease in interpersonal setting compared to the baseline in controls but not in ASD group. Caffeine intake, smoking, exercise, or psychiatric comorbidities did not influence these results. It might be conceptualised as a floor effect; the overall decreased flexibility of vagal tone or parasympathetic regulation leaves no room for further reduction in ASD. This confirms previous research findings of decreased regulatory capacity of participants with ASD in social situations.

Additionally, we calculated HRV during the social distance regulation task in 10 s time periods, applying ultra-short-term analysis [61] exactly at the time point when participants arrived at the reported distance in order to take a closer look at the relationship between interpersonal distance and autonomic regulation. To test the more nuanced aspects of interpersonal distance regulation experimentally, we assessed the modulatory effect of eye contact and attribution. We did not find an effect of the modulatory factors regarding the 10 s HRV metrics, and the results did not directly support our hypothesis that HRV predicts interpersonal distance. Nevertheless, we can speculate that due to the inherently lower baseline HRV in ASD, the diminished capacity of reactive decrement might have prevented further fine-tuning during the interpersonal task. Reduced regulatory capacity, combined with elevated amygdala reactivity could lead to early exhaustion even during minimal social interaction. This can raise the possibility that the larger interpersonal distance is the consequence of the early exhaustion of regulatory capacity, and by keeping the distance they might avoid a more severe autonomic disturbance in social situations. To test this hypothesis in real-life situations further, applying widely available wearable devices might be useful. The experience we had gained could be used later, for example, to develop biofeedback tools for social communication training for autistic people.

### Limitations and further directions

Despite the most careful planning, every study has its limitations. In this study, we examined adult participants with ASD to measure interpersonal distance and autonomic regulation simultaneously. We recruited participants with average or above-average intellectual abilities, which increases the likelihood of adaptive skill acquisition. To overcome this limitation, the inclusion of a broader spectrum of autistic participants is needed in future studies.

We found greater interpersonal distance in ASD, measured by the modified version of the stop distance paradigm, but there was no difference between study groups regarding heart rate variability during that part of the experiment. In subsequent research, HRV differences should be measured at fixed distances as well (even closer and farther than comfortable). Subjective rating of the level of comfort (both by participants and by the experimenter) might help to gain a better insight into how correctly the experimenter’s perspective can be estimated by the participants.

In this study the experimenter was female regardless of the gender of the participants, and also a person unknown to the participants. Further studies may also require the testing of different gender pairs of examinees. Involving people who are familiar and present in the life of the participants also can be useful.

Unfortunately, our data collection took place in 2019–2020, and due to COVID-19, we were unable to collect data with the original study design, especially given that the pandemic significantly impacted the scope of this study (interpersonal distance). Thus, it was essential to check the achieved statistical power to make sure whether it limits the interpretation of our results. We found that regarding the interpersonal distance, all analyses but one (the eye contact × attribution × group interaction) achieved sufficient power. In the interpersonal HRV analysis, most effects were seriously underpowered, as well as the group main effects in the HR and HRV analyses. To test whether the nonsignificant results in these cases were due to the low power or the nonexistence of these effects, we conducted Bayesian analyses as well. We found that the underpowered effects are unlikely to benefit the model, both in the interpersonal distance and interpersonal HRV analyses. The HR and HRV baseline/experiment analysis, the group main effects did not achieve sufficient statistical power either (for details see Table 2 and the S3 Table). Taken together, the focus of our study (the interpersonal distance and the interpersonal HRV) either had a sufficient power to detect the effect or they were likely to be null results indeed. However, further studies are needed to address whether HR and HRV per se differ in ASD and neurotypical individuals.

Further studies should measure the different effect on preferred interpersonal distance in ASD since for a longer period of time, a recommended distance has been regularly and explicitly proposed. Additional conditions with and without wearing face masks might be considered, too. These subsequent studies will be able to show us whether autistic people have been affected differently than neurotypicals by social distancing measures.

## Conclusion

Interpersonal distance regulation is a relevant nonverbal part of social communication. It reflects the individual need for personal space and the ability to read others’ intentions. Together with other biomarkers of autonomic functions, this might express how demanding a simple social interaction can be for people with ASD. In this study, we introduced a new experimental design to measure these factors together in a basic social interaction setting. Although (predominantly male) adults with autism preferred greater interpersonal distance from a female and had higher heart rates compared to males without autism, and participants with ASD had lower baseline heart rate variability and decreased heart rate variability reactivity than controls, there was overlap in the distributions of the two groups. We failed to detect significant modulatory effects of eye contact and attribution (the prediction of the experimenter’s preferred distance) in both study groups. The results that the modulatory factors we chose did not show unequivocal influence were contrary to our expectations. Although both groups presented a reduced HRV during the interpersonal distance task compared to baseline, the decrease was less evident in the ASD group. We cannot exclude the possibility that this might be due to the fact that participants with ASD already had a reduced HRV at baseline compared to control participants, rather than altered regulatory processes during the interpersonal distance task. We believe that applying this experimental design supplemented with lessons learned could also be beneficial in studying other psychiatric conditions, such as borderline personality disorder, anxiety, social phobia, or psychosis. Further studies are recommended to grasp the complexity and underlying factors of distance regulation in typical and atypical populations. These findings may further expand our understanding of interpersonal distance regulation and help to disentangle what is due to autism and what is a consequence of a potential comorbid psychiatric condition.

## Supporting information

S1 Table Social distance in intentional conditions. Descriptive statistics and group differences. ASD: Autism Spectrum Disorder, NTP: Neurotypical Participant, N: sample size, SD: standard deviation.
(DOCX)

S2 Table HRV in explicit conditions. Descriptive statistics and group differences. ASD: Autism Spectrum Disorder, NTP: Neurotypical Participant, N: sample size, SD: standard deviation.
(DOCX)

S3 Table Statistical power of the relevant effects. (DOCX)

S4 Table Bayesian analyses: Model comparisons. (DOCX)

S1 File Questionnaires and Posthoc power analyses. (DOCX)
