# Supplementary material for: Altered interpersonal distance regulation in autism spectrum disorder
Source: PLoS One. 2023 Mar 31;18(3):e0283761. doi: 10.1371/journal.pone.0283761 (PMC10065277; doi:10.1371/journal.pone.0283761)
Supplement: S3 Table — (DOCX) [file pone.0283761.s003.docx]

**S3 Table. Statistical power of the relevant effects**

| **Effect** | **Post hoc achieved power** | **Sufficiency of power** |
| --- | --- | --- |
| **Interpersonal distance** | | |
| group | 95.89% | sufficient |
| eye contact × group | 100% | sufficient |
| attribution × group | 99.54% | sufficient |
| eye contact × attribution × group | 6.45% | underpowered |
| **HR baseline vs experiment** | | |
| group | 10.78% | underpowered |
| group × time | 98.15% | sufficient |
| **HRV baseline vs experiment** | | |
| group | 24.23% | underpowered |
| group × time | 100.00% | sufficient |
| **HRV in interpersonal situation** | | |
| group | 5.00% | underpowered |
| eye contact × group | 88.14% | sufficient |
| attribution × group | 45.66% | underpowered |
| eye contact × attribution × group | 49.13% | underpowered |
