# Supplementary material for: Altered interpersonal distance regulation in autism spectrum disorder
Source: PLoS One. 2023 Mar 31;18(3):e0283761. doi: 10.1371/journal.pone.0283761 (PMC10065277; doi:10.1371/journal.pone.0283761)
Supplement: S4 Table — (DOCX) [file pone.0283761.s004.docx]

**S4 Table. Bayesian analyses: model comparisons.**

| **Interpersonal distance** | | |
| --- | --- | --- |
| **Models** | **BF_01_** | **error %** |
| Null model (incl. subject and random slopes) | 1 |  |
| Group | 0.147 | 5.809 |
| Eye contact + Group | 0.191 | 4.409 |
| Eye contact + Group + Eye contact * Group | 0.253 | 5.161 |
| Attribution + Group | 0.489 | 12.06 |
| Eye contact + Attribution + Group | 0.499 | 15.687 |
| Eye contact + Attribution + Group + Eye contact * Attribution + Attribution * Group | 0.551 | 20.619 |
| Eye contact + Attribution + Group + Eye contact * Attribution | 0.606 | 17.373 |
| Attribution + Group + Attribution * Group | 0.614 | 13.153 |
| Eye contact + Attribution + Group + Eye contact * Group | 0.667 | 13.8 |
| Eye contact + Attribution + Group + Eye contact * Attribution +  Eye contact * Group | 0.83 | 19.79 |
| Eye contact + Attribution + Group + Attribution * Group | 0.84 | 14.195 |
| Eye contact | 1.306 | 2.802 |
| Eye contact + Attribution + Group + Eye contact * Attribution +  Eye contact * Group + Attribution * Group | 1.332 | 17.158 |
| Eye contact + Attribution + Group + Eye contact * Group + Attribution * Group | 1.396 | 16.353 |
| Attribution | 2.445 | 11.38 |
| Eye contact + Attribution + Group + Eye contact * Attribution + Eye contact * Group + Attribution * Group + Eye contact * Attribution * Group | 4.254 | 16.372 |
| Eye contact + Attribution | 4.385 | 14.944 |
| Eye contact + Attribution + Eye contact * Attribution | 4.436 | 17.162 |
|  | | |
| **HR baseline vs experiment** | | |
| **Models** | **BF_01_** | **error %** |
| Null model (incl. subject and random slopes) | 1 |  |
| Time | 4.17E-05 | 0.916 |
| Time + Group | 5.16E-05 | 9.081 |
| Time + Group + Time * Group | 1.41E-04 | 4.141 |
| Group | 1.44 | 2.002 |
|  | | |
|  | | |
|  | | |
| **HRv baseline vs experiment** | | |
| **Models** | **BF_01_** | **error %** |
| Null model (incl. subject and random slopes) | 1 |  |
| Time + Group + Time * Group | 0.001 | 1.98 |
| Time + Group | 0.002 | 2.228 |
| Time | 0.003 | 0.999 |
| Group | 0.783 | 1.129 |
|  | | |
| **HRV interpersonal** | | |
| **Models** | **BF_01_** | **error %** |
| Null model (incl. subject and random slopes) | 1 |  |
| Group | 2.268 | 12.536 |
| Eye contact | 2.304 | 1.507 |
| Attribution | 3.311 | 1.419 |
| Eye contact + Attribution | 7.314 | 4.293 |
| Attribution + Group | 7.666 | 14.883 |
| Eye contact + Group | 7.871 | 13.996 |
| Eye contact + Group + Eye contact * Group | 11.924 | 13.439 |
| Eye contact + Attribution + Group | 14.368 | 13.872 |
| Attribution + Group + Attribution * Group | 14.821 | 14.433 |
| Eye contact + Attribution + Eye contact * Attribution | 27.232 | 4.05 |
| Eye contact + Attribution + Group + Attribution * Group | 28.827 | 18.671 |
| Eye contact + Attribution + Group + Eye contact * Group | 33.108 | 17.517 |
| Eye contact + Attribution + Group + Eye contact * Attribution | 59.799 | 17.327 |
| Eye contact + Attribution + Group + Eye contact * Group + Attribution * Group | 65.947 | 24.78 |
| Eye contact + Attribution + Group + Eye contact * Attribution + Attribution * Group | 107.221 | 18.387 |
| Eye contact + Attribution + Group + Eye contact * Attribution +  Eye contact * Group | 156.539 | 21.723 |
| Eye contact + Attribution + Group + Eye contact * Attribution +  Eye contact * Group + Attribution * Group | 435.216 | 17.101 |
| Eye contact + Attribution + Group + Eye contact * Attribution + Eye contact * Group + Attribution * Group + Eye contact * Attribution * Group | 892.745 | 22.036 |

*Note*.  All models include subject, and random slopes for all repeated measures factors.
