# Supplementary material for: Altered interpersonal distance regulation in autism spectrum disorder
Source: PLoS One. 2023 Mar 31;18(3):e0283761. doi: 10.1371/journal.pone.0283761 (PMC10065277; doi:10.1371/journal.pone.0283761)
Supplement: S1 File — (DOCX) [file pone.0283761.s005.docx]

**Supplementary material**

**Questionnaires**

**1.** **Autism-Spectrum Quotient (AQ).** To assess autistic traits in our sample, we used the AQ50 (Simon Baron-Cohen et al., 2001). The questionnaire consists of 50 questions about e.g. social skills, communication, or attention to details. Participants had to rate the items on a four-point Likert scale (1-4, indicating “definitely disagree”, “slightly disagree”, “slightly agree”, and “definitely agree”, respectively). When scoring the questionnaire, these options were dichotomized, all agreed answers were scored as 1, the other two options as 0 in non-reversed items, the opposite in the case of reverse items. Thus, the maximum score of the questionnaire was 50 (the clinical threshold for ASD >=32). (Chronbach’s alpha in our study = 0.92)

**2. Mentalization Questionnaire (MZQ).** Mentalization abilities were measured by the MZQ (Hausberg et al., 2012), which is a 15-item self-report questionnaire. MZQ has four subscales: ‘Refusing self-reflection’, ‘Emotional awareness’, ‘Psychic equivalence mode’ and ‘Regulation of affect’, rating on a five-point scale, (from “strongly disagree” to “strongly agree”). The total score ranges from 15 to 75 (indicating from good to poor mentalization skills, respectively). According to Hausberg et al. (2012), (n=434), the Cronbach’s alpha of the MZQ total score is 0.81, which can be considered as good consistency, but internal consistencies for the subscales ranged from 0.54 (Regulation of affect), to 0.72 (Psychic equivalence mode) among neurotypical participants which have been considered insufficient to satisfactory according to the COTAN criteria (Paridaens, 2016). For this reason, we use the total score in this study. (Chronbach’s alpha = 0.81)

**3. Adult ADHD Self-Report Scale v1.1 (ASRS).** The ASRS is a questionnaire that evaluates ADHD symptoms as defined in the Diagnostic and Statistical Manual of Mental Disorders fourth edition (DSM-IV) (Silverstein et al., 2019). ASRS is divided into two parts: Part A (consisting of 6 questions) and Part B (12 questions). For each item, respondents are asked to indicate how often the stated symptom occurred over the prior six months, with five options: never, rarely, sometimes, often, or very often. Part B items provide insight into the frequency of symptoms and can be used to help elicit other symptoms the patient may suffer from. According to Silverstein et al. (2019), the reliability of the ASRS and subscales is high (Cronbach’s α >= 0.84). In our study Cronbach’s alpha (total) = 0.87, part A = 0.61, part B = 0.85)

**4. Adult Attachment Scale (AAS).** We used the Dependence and Anxiety subscales of the AAS (Collins, 1996) to measure the attachment style of our participants. The questionnaire consists of 18 items, with three subscales. Participants rated on a scale from one (“Not typical to me at all”) to five (“Very typical to me”) how they think they function/would function in a romantic relationship. Collins et al. reported Cronbach's alpha coefficients of 0.69 for close, 0.75 for depend, and 0.72 for anxiety subscale (Collins & Read, 1990). Later authors found a slightly modified structure more useful and consistent. The scale measures adult attachment styles named *secure* (high scores on close and depend subscales, low score on anxiety subscale), *anxious* (high score on anxiety subscale, moderate scores on close and depend subscales), and *avoidant* (low scores on close, depend, and anxiety subscales) (Collins, 1996). We decided to use two subscales (anxious and avoidant) of attachment style in this study (Chronbach’s alpha total = 0.88, anxious = 0.85, avoidant = 0.85).

**5. State-Trait Anxiety Inventory (STAI).** STAI is a commonly used measure of trait and state anxiety (Spielberger et al., 1983). It contains 20 items for assessing state and trait anxiety. All items are rated on a four-point scale (from “Almost Never” to “Almost Always”). Higher scores indicate greater anxiety. Internal consistency coefficients for the scale have ranged from .86 to .95; test-retest reliability coefficients have ranged from .65 to .75 over a 2-month interval (Spielberger et al., 1983). In our study, we measured only trait anxiety. Chronbach’s alpha = 0.92).

**Posthoc power analyses**

To test whether our analyses achieved a sufficient statistical power, we ran post hoc power analyses using G*Power 3.1.9.7 (Faul et al., 2007). We determined the effect size *f*s based on either the *η^2^_p_* reported in the main article (in the case of interactions) or the means, standard deviations and Ns (in the case of group main effects). We considered power above 80% sufficient. Furthermore, since some analyses yielded nonsignificant results, we performed Bayesian analyses to test whether the nonsignificance is more likely to reflect a true null result or the lack of statistical power (see in the article and supplementary material).

The performed post hoc power analyses confirmed that our data was sufficient to find an existing effect regarding the interpersonal distance. The only exception was the eye contact × attribution × group interaction. The analyses on the interpersonal HRV were underpowered (with the exception of the eye contact × group interaction). In the HR and HRV baseline/experiment analysis, the group main effects did not achieve a sufficient statistical power, but the interactions between time and group did.

**References**

Baron-Cohen, S., Wheelwright, S., Skinner, R., Martin, J., & Clubley, E. (2001). The Autism-Spectrum Quotient (AQ): Evidence from Asperger Syndrome/High-Functioning Autism, Malesand Females, Scientists and Mathematicians. Journal of Autism and Developmental Disorders, 31(1), 5–17. <https://doi.org/10.1023/A:1005653411471>

Faul, F., Erdfelder, E., Lang, A. G., & Buchner, A. (2007). G*Power 3: A flexible statistical power analysis program for the social, behavioral, and biomedical sciences. *Behavior Research Methods*, *39*(2), 175–191. <https://doi.org/10.3758/BF03193146/METRICS>

Hausberg, M. C., Schulz, H., Piegler, T., Happach, C. G., Klöpper, M., Brütt, A. L., Sammet, I., & Andreas, S. (2012). Is a self-rated instrument appropriate to assess mentalization in patients with mental disorders? Development and first validation of the Mentalization Questionnaire (MZQ). Psychotherapy Research, 22(6), 699–709. <https://doi.org/10.1080/10503307.2012.709325>

Paridaens, P. (2016). Reliability and validity of the Mentalization Questionnaire (MZQ) in forensic care. 39.

Silverstein, M. J., Faraone, S. V., Alperin, S., Leon, T. L., Biederman, J., Spencer, T. J., & Adler, L. A. (2019). Validation of the Expanded Versions of the Adult ADHD Self-Report Scale v1.1 Symptom Checklist and the Adult ADHD Investigator Symptom Rating Scale. Journal of Attention Disorders, 23(10), 1101–1110. <https://doi.org/10.1177/1087054718756198>

Spielberger, C. D., Gorsuch, R. L., Lushene, R., Vagg, P. R., & Jacobs, P. A. (1983). Manual for the State-Trait Anxiety Inventory. Palo Alto, CA: Consulting Psychologists Press.
